# Triglyceride glucose index is an independent predictor for the progression of coronary artery calcification in the absence of heavy coronary artery calcification at baseline

**DOI:** 10.1186/s12933-020-01008-5

**Published:** 2020-03-16

**Authors:** Ki-Bum Won, Eun Ji Park, Donghee Han, Ji Hyun Lee, Su-Yeon Choi, Eun Ju Chun, Sung Hak Park, Hae-Won Han, Jidong Sung, Hae Ok Jung, Hyuk-Jae Chang

**Affiliations:** 1 Division of Cardiology, Ulsan University Hospital, University of Ulsan College of Medicine, Ulsan, South Korea; 2 Division of Cardiology, Severance Cardiovascular Hospital, Yonsei University College of Medicine, Yonsei University Health System, Seoul, South Korea; 3 Medical Information Center, Ulsan University Hospital, Ulsan, South Korea; 4 Division of Cardiology, New York-Presbyterian Hospital and Weill Cornell Medical College, New York, NY USA; 5 Division of Cardiology, Myongji Hospital, Ilsan, South Korea; 6 Division of Cardiology, Healthcare System Gangnam Center, Seoul National University Hospital, Seoul, South Korea; 7 Division of Radiology, Seoul National University Bundang Hospital, Seongnam, South Korea; 8 Division of Radiology, Gangnam Heartscan Clinic, Seoul, South Korea; 9 Department of Internal Medicine, Gangnam Heartscan Clinic, Seoul, South Korea; 10 Division of Cardiology, Heart Stroke & Vascular Institute, Samsung Medical Center, Seoul, South Korea; 11 Division of Cardiology, Seoul St. Mary’s Hospital, College of Medicine, The Catholic University of Korea, Seoul, South Korea; 12 Division of Cardiology, Severance Cardiovascular Hospital, Yonsei-Cedars-Sinai Integrative Cardiovascular Imaging Research Center, Yonsei University College of Medicine, Yonsei University Health System, 50-1 Yonsei-ro, Seodaemun-gu, Seoul, 03722 South Korea

**Keywords:** Triglyceride glucose index, Insulin resistance, Coronary artery calcification, Atherosclerosis

## Abstract

**Background:**

Data on the relationship between the triglyceride glucose (TyG) index and coronary artery calcification (CAC) progression is limited. This longitudinal study evaluated the association of TyG index with CAC progression in asymptomatic adults.

**Methods:**

We enrolled 12,326 asymptomatic Korean adults who had at least two CAC evaluations. The TyG index was determined using ln (fasting triglycerides [mg/dL] × fasting glucose [mg/dL]/2). CAC progression was defined as a difference ≥ 2.5 between the square roots (√) of the baseline and follow-up coronary artery calcium score (CACS) (Δ√transformed CACS). Annualized Δ√transformed CACS was defined as Δ√transformed CACS divided by the inter-scan period.

**Results:**

During a mean 3.3 years, the overall incidence of CAC progression was 30.6%. The incidence of CAC progression (group I [lowest]: 22.7% versus [vs.] group II: 31.7% vs. group III [highest]: 37.5%, P < 0.001) and annualized Δ√transformed CACS (group I: 0.46 ± 1.44 vs. group II: 0.71 ± 2.02 vs. group III: 0.87 ± 1.75, P < 0.001) were markedly elevated with increasing TyG index tertiles. Multivariate linear regression analysis showed that TyG index was associated with annualized Δ√transformed CACS (β = 0.066, P = 0.036). In multivariate logistic regression analysis, the TyG index was significantly associated with CAC progression in baseline CACS ≤ 100.

**Conclusion:**

The TyG index is an independent predictor of CAC progression, especially in adults without heavy baseline CAC.

## Background

Recent epidemiologic study revealed that the number of people who died from cardiovascular (CV) disease in 2013 was more than 17.3 million, representing an increase from 1990 of 40.8% [1]. The progression of atherosclerosis-related CV disease (ASCVD) is strongly associated with the risk of CV morbidity and mortality. Coronary artery calcification (CAC) is an effective marker for ASCVD and predicts adverse clinical outcomes [2–4]. Thus, CV risk is commonly assessed by the coronary artery calcium score (CACS), which is determined by computed tomography. In addition to baseline CACS and conventional CV risk factors, CAC progression is known as a powerful predictor of mortality [5].

The triglyceride glucose (TyG) index has been suggested as a reliable surrogate marker of insulin resistance (IR), which is a substantial risk factor for ASCVD [6–9]. Previous studies reported that TyG index is significantly associated with an increased risk of developing type 2 diabetes, hypertension, and adverse CV events [10–14]. In addition, several cross-sectional studies identified the strong relation between the TyG index and atherosclerosis in different clinical conditions [15–21]. However, data on the association of TyG index with CAC change in asymptomatic adults is limited. Especially, the significance of TyG index on CAC progression based on baseline CAC status has not been evaluated in a large population. Therefore, in the present study, we evaluated the association between the TyG index and CAC progression in asymptomatic 12,326 Korean adults.

## Methods

### Study population and design

Data from the Korea Initiatives on Coronary Artery Calcification (KOICA) registry were analysed in the present study. Briefly, the KOICA is a retrospective, multicentre, and observational registry with single ethnicity in the setting of self-referral for asymptomatic subjects who underwent general health examination at six healthcare centres in South Korea [22]. Overall, 93,707 subjects were enrolled in the KOICA registry from December 2012 to August 2016. Self-reported medical questionnaires were used to obtain information on previous medical history. All data were obtained during visits to each healthcare centre. Among the 93,707 subjects from this registry, 12,326 subjects who underwent at least two CAC scan examinations with available data on the TyG index and diabetic status were finally included in the present study.

### Data collection

Information on history of hypertension, diabetes, hyperlipidaemia, and smoking status for each subject was systematically collected. Weight, height, and blood pressure were measured during the healthcare centre visits. Weight and height were measured during the subjects wore light clothing without shoes. The body mass index was calculated as weight (kg)/height (m^2^). Blood pressure of the right arm was measured using an automatic manometer after resting for at least more than 5 min. All blood samples including total cholesterol, triglyceride, high-density lipoprotein cholesterol (HDL-C), low-density lipoprotein cholesterol (LDL-C), creatinine, glucose, and glycated haemoglobin A1C (HbA1C) levels were obtained after at least 8 h of fasting and analysed. All methods were performed in accordance with the relevant guidelines and regulations.

### Ethical statement

The appropriate institutional review board committees of each centre approved the protocol of present study.

### Definitions

Hypertension was defined as systolic blood pressure ≥ 140 mmHg or diastolic blood pressure ≥ 90 mmHg, a previous diagnosis of hypertension, or use of anti-hypertensive medication [23]. Hyperlipidaemia was defined as a total cholesterol level of ≥ 240 mg/dL or anti-hyperlipidemic treatment. Diabetes was defined as either a fasting glucose level ≥ 126 mg/dL, HbA1C level ≥ 6.5%, a referral diagnosis of diabetes, or use of anti-diabetic treatment [24]. Current smoking history was considered present if subjects currently smoked or smoked until 1 month before the study. The TyG index was determined using ln (triglycerides [mg/dL] × glucose [mg/dL]/2). All participants were categorised into three groups based on the tertile of the TyG index level.

CACS was measured based on the scoring system, as previously described by Agatston et al. [25]. CAC progression was defined as a difference ≥ 2.5 between the square roots (√) of the baseline and follow-up CACSs (Δ√transformed CACS) considering inter-scan variability [26]. Annualised Δ√transformed CACS was defined as Δ√transformed CACS divided by the inter-scan period. All computed tomography (CT) scans used to assess CAC were obtained from a > 16-slice multi-detector CT scanner (GE 64-slice Lightspeed, Siemens 16-slice Sensation, Philips Brilliance 256 iCT, and Philips Brilliance 40-channel multi-detector CT). All centres performed standard prospective or retrospective methods.

### Statistical analysis

Continuous variables are expressed as the mean ± standard deviation. Categorical variables are presented as absolute values and proportions. After checking the distribution status of variables, the one-way analysis of variance test was used to analyse continuous variables and the χ^2^ or Fisher’s exact test was used to analyse categorical variables, as appropriate. Multivariate linear regression analysis was used for the association of clinical variables with annualized Δ√transformed CACS in overall participants. Multivariate logistic regression analysis was performed to identify independent predictors for CAC progression according to categorical baseline CACS. All statistical analyses were performed using the Statistical Package for the Social Sciences version 19 (SPSS, Chicago, Illinois) and SAS version 9.1.3 (SAS Institute Inc., Cary, North Carolina). A P-value < 0.05 was considered significant in all analyses.

## Results

### Baseline characteristics

The mean age of the 12,326 participants (10,382 men, 84.2%) was 51.7 ± 8.5 years. The prevalence of hypertension, diabetes, hyperlipidaemia, and current smoking was 33.5%, 13.8%, 28.0%, and 28.5%, respectively. The ranges of the TyG index in stratified groups of I (lowest), II, and III (highest) were 6.77–8.40, 8.41–8.91, and 8.92–11.62, respectively. Systolic and diastolic blood pressure; body mass index (BMI); the levels of total cholesterol, triglyceride, LDL-C, glucose, HbA1C, and creatinine; and the prevalence of male sex, hypertension, diabetes, hyperlipidaemia, and current smoking significantly increased with increasing TyG index tertiles. In contrast, the mean age and HDL-C levels significantly decreased with increasing tertiles (Table 1).

**Table 1:** Clinical characteristics of the study cohort

	Total (n = 12,326)	Tertile of TyG index			
		I (lowest) (n = 4117) 6.77–8.40	II (n = 4129) 8.41–8.91	III (highest) (n = 4080) 8.92–11.62	P
Age, years	51.7 ± 8.5	51.4 ± 9.0	52.1 ± 8.4	51.6 ± 8.0	< 0.001
Male, n (%)	10,382 (84.2)	2974 (72.2)	3633 (88.0)	3775 (92.5)	< 0.001
Systolic BP, mmHg	119.6 ± 15.0	117.1 ± 15.0	119.8 ± 14.9	121.8 ± 14.8	< 0.001
Diastolic BP, mmHg	75.0 ± 10.5	72.9 ± 10.7	75.2 ± 10.2	77.1 ± 10.3	< 0.001
BMI, kg/m^2^	24.6 ± 2.8	23.5 ± 2.7	24.6 ± 2.6	25.5 ± 2.6	< 0.001
Hypertension, n (%)	4016 (33.5)	1036 (26.2)	1355 (33.8)	1625 (40.8)	< 0.001
Diabetes, n (%)	1703 (13.8)	248 (6.0)	490 (11.9)	965 (23.7)	< 0.001
Hyperlipidemia, n (%)	3455 (28.0)	726 (17.6)	1102 (26.7)	1627 (39.9)	< 0.001
Current smoking, n (%)	3229 (28.5)	747 (19.9)	1086 (28.6)	1396 (37.0)	< 0.001
Total cholesterol, mg/dL	197.5 ± 34.0	188.2 ± 31.7	197.5 ± 32.9	206.8 ± 34.9	< 0.001
Triglyceride, mg/dL	141.7 ± 89.4	73.2 ± 17.3	122.6 ± 22.2	230.0 ± 102.2	< 0.001
HDL-C, mg/dL	53.3 ± 16.0	60.2 ± 16.4	52.7 ± 14.9	47.1 ± 13.6	< 0.001
LDL-C, mg/dL	122.0 ± 31.7	114.2 ± 29.5	125.5 ± 31.1	126.2 ± 33.1	< 0.001
Glucose, mg/dL	97.8 ± 20.3	89.5 ± 10.7	96.4 ± 14.0	107.6 ± 27.7	< 0.001
HbA1C, %	5.7 ± 0.7	5.5 ± 0.5	5.6 ± 0.6	5.9 ± 1.0	< 0.001
Creatinine, mg/dL	0.95 ± 0.17	0.92 ± 0.18	0.96 ± 0.16	0.97 ± 0.17	< 0.001

Values are presented as the mean ± standard deviation or number (%)

*BMI* body mass index, *BP* blood pressure, *HbA1C* glycated haemoglobin A1C, *HDL*-*C* high-density lipoprotein cholesterol, *LDL*-*C* low-density lipoprotein cholesterol, *TyG* triglyceride glucose

### Baseline and change of CAC

**Fig. 1:**
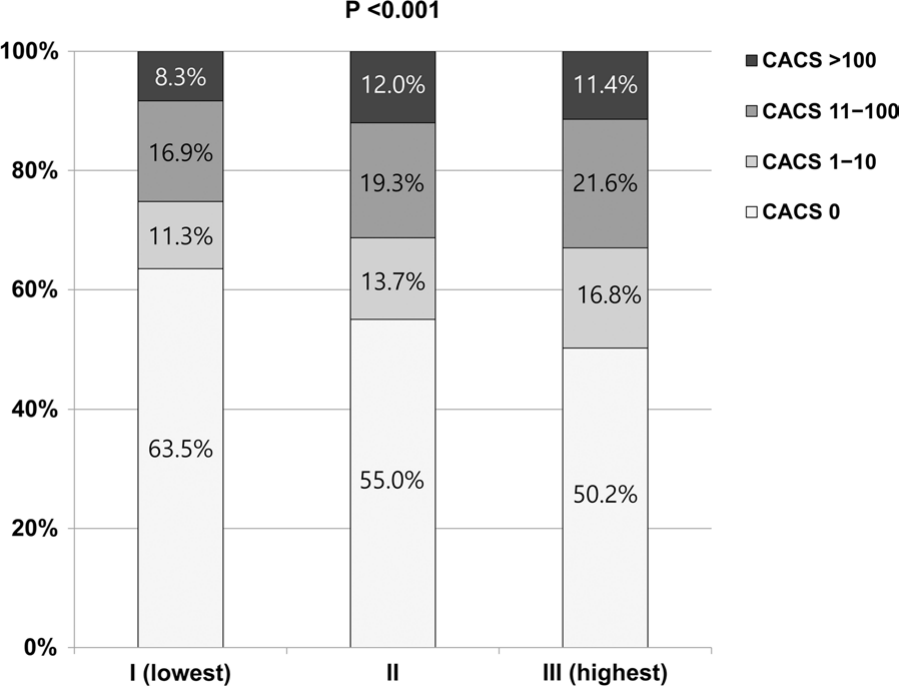
Comparison of baseline coronary artery calcification according to triglyceride glucose index tertiles. *CACS* coronary artery calcium scores

**Fig. 2:**
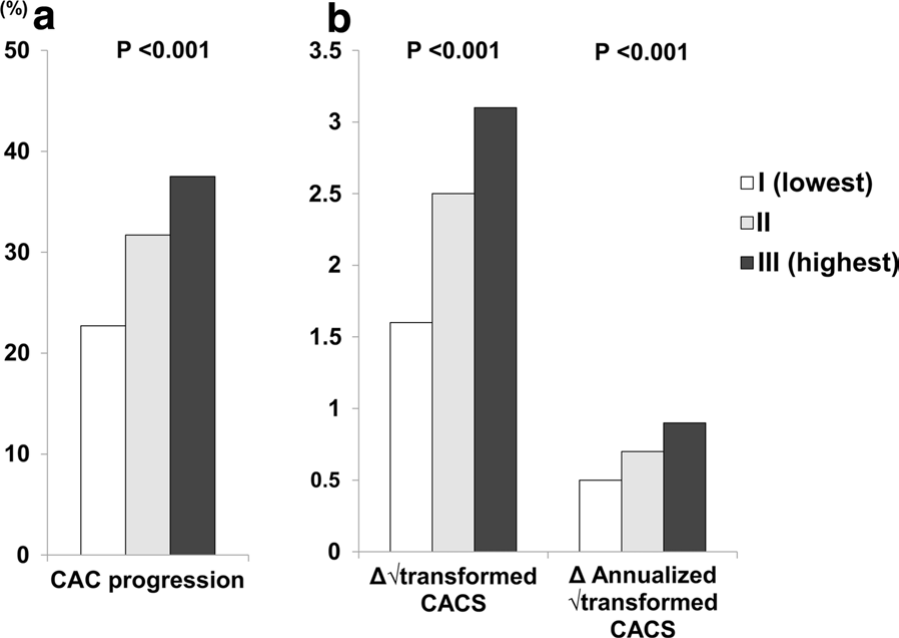
Changes of CAC according to TyG index tertiles. *CAC* coronary artery calcification, *TyG* triglyceride glucose

At baseline, overall prevalence of CACA 0, 1–10, 11–100, and > 100 was 56.2%, 13.9%, 19.3%, and 10.6%, respectively. According to the TyG index tertiles, the mean CACSs were 36.8 ± 153.9, 50.8 ± 174.4, and 50.0 ± 186.1 in group I, group II, and group III, respectively. The significant difference in the proportion of categorical CACS among the three groups was observed (Fig. 1). During the mean inter-scan period of 3.3 ± 1.8 years, the overall incidence of CAC progression was 30.6%. The incidence of CAC progression (group I: 22.7% versus [vs.] group II: 31.7% vs. group III: 37.5%, P < 0.001), Δ√transformed CACS (group I: 1.65 ± 3.95 vs. group II: 2.52 ± 4.85 vs. group III: 3.09 ± 5.21, P < 0.001), and annualised Δ√transformed CACS (group I: 0.46 ± 1.44 vs. group II: 0.71 ± 2.02 vs. group III: 0.87 ± 1.75, P < 0.001) were elevated with increasing TyG index tertiles (Fig. 2).

### TyG index and CAC change in each categorical CACS

**Fig. 3:**
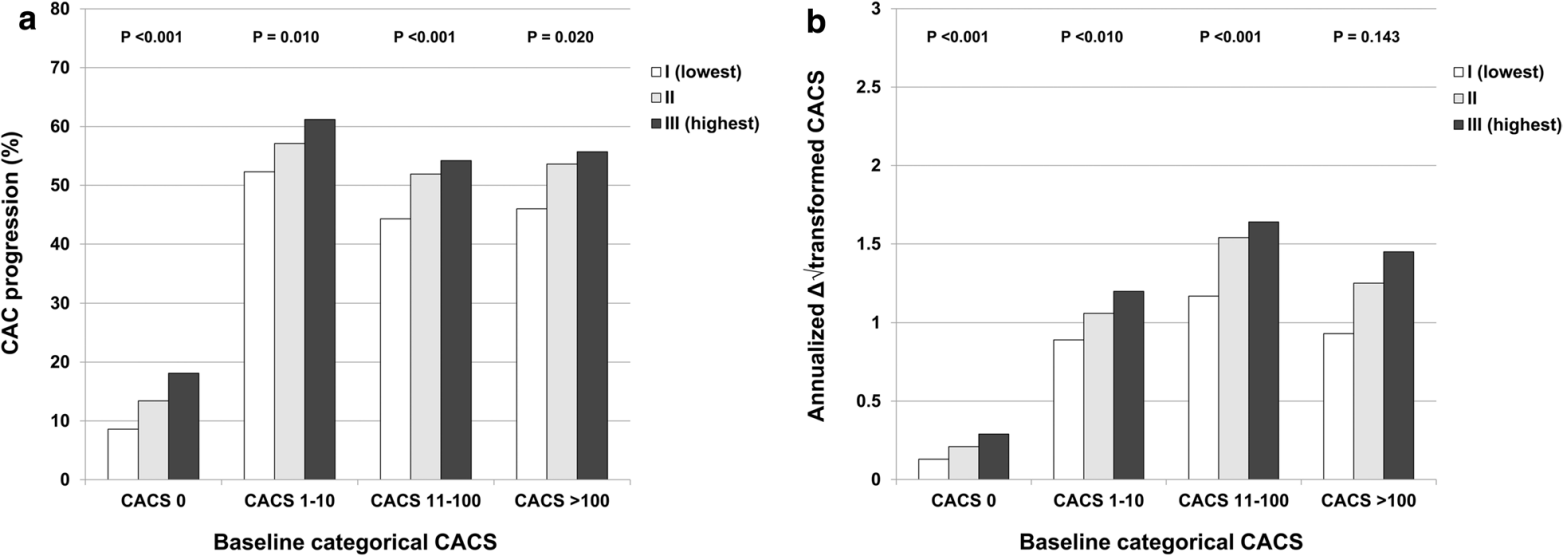
Changes of CAC according to the baseline CACS. *CAC* coronary artery calcification, *CACS* coronary artery calcium score

The incidence of CAC progression was elevated with increasing TyG index tertiles in categorical CACSs of 0, 1–10, 11–100, and > 100 (CACS 0: 8.6% vs. 13.4% vs. 18.1%, P < 0.001; CACS 1–10: 52.3% vs. 57.1% vs. 61.2%, P < 0.001; CACS 11–100: 44.3% vs. 51.9% vs. 54.2%, P < 0.001; CACS > 100: 46.0% vs. 53.6% vs. 55.7%, P = 0.020). Annualised Δ√transformed CACS was elevated with increasing TyG index tertiles in categorical CACSs of 0 (0.13 ± 0.48 vs. 0.21 ± 0.56 vs. 0.29 ± 0.85, P < 0.001), 1–10 (0.89 ± 1.07 vs. 1.06 ± 1.26 vs. 1.20 ± 1.24, P < 0.001), and 11–100 (1.17 ± 1.87 vs. 1.54 ± 2.30 vs. 1.64 ± 2.26, P < 0.001), not in CACS > 100 (0.93 ± 3.51 vs. 1.25 ± 4.44 vs. 1.45 ± 2.93, P = 0.143) (Fig. 3).

### Association between clinical variables and CAC change

Multivariate linear regression analysis showed that age, male sex, hypertension, diabetes, hyperlipidaemia, obesity, current smoking, creatinine, TyG index, and baseline CACS > 100 were associated with annualized Δ√transformed CACS (All P < 0.05) (Table 2). The result of multivariate logistic regression analysis for the association between clinical variables and CAC progression was described in Additional file 1: Table S1.

**Table 2:** Association of clinical variables with annualized Δ√transformed CACS

Variables	β	SE	P
Age, pre-1 year increase	0.032	0.002	< 0.001
Male	0.281	0.055	< 0.001
Hypertension	0.255	0.037	< 0.001
Diabetes	0.334	0.050	< 0.001
Hyperlipidemia	0.168	0.037	< 0.001
Obesity	0.113	0.035	0.001
Current smoking	0.146	0.037	< 0.001
Creatinine, per-1 mg/dL increase	0.357	0.111	0.001
TyG index, per-1 increase	0.066	0.031	0.036
Baseline CACS > 100	0.230	0.057	< 0.001

*BMI* body mass index, *CACS* coronary artery calcium score, *TyG* triglyceride glucose

### TyG index and the risk of CAC progression according to the baseline CACS

With regards to the relation of TyG index with CAC progression according to the baseline CACS, the TyG index (per 1-unit increase) was associated with an increased risk of CAC progression in baseline CACSs of 0, 1–10, and 11–100 after adjusting for age, male sex, BMI, hypertension, diabetes, hyperlipidaemia, current smoking, and serum creatinine level. However, this association of the TyG index and CAC progression was not observed in the condition with baseline CACS > 100 (Table 3). After adjusting for age, male sex, BMI, systolic and diastolic BP, the level of total cholesterol, triglyceride, HDL-C, LDL-C, glucose, and creatinine, and current smoking, the relation of TyG index and CAC progression was consistent (Additional file 2: Table S2).

**Table 3:** Impact of the TyG index (per 1-unit increase) on CAC progression based on baseline categorical CACS

	OR (95% CI)	P	RR (95% CI)	P
CACS 0				
Model 1	1.83 (1.63–2.05)	< 0.001	1.67 (1.52–1.82)	< 0.001
Model 2	1.37 (1.18–1.59)	< 0.001	1.30 (1.15–1.46)	< 0.001
CACS 1–10				
Model 1	1.39 (1.17–1.65)	< 0.001	1.15 (1.07–1.23)	< 0.001
Model 2	1.28 (1.05–1.57)	0.016	1.11 (1.02–1.20)	0.014
CACS 11–100				
Model 1	1.42 (1.24–1.64)	< 0.001	1.18 (1.11–1.26)	< 0.001
Model 2	1.34 (1.14–1.59)	0.001	1.15 (1.06–1.24)	< 0.001
CACS > 100				
Model 1	1.33 (1.09–1.62)	0.004	1.14 (1.04–1.24)	0.003
Model 2	1.04 (0.83–1.31)	0.743	1.02 (0.92–1.14)	0.702

Models: 1 = unadjusted; 2 = adjusted for age, male sex, BMI, hypertension, diabetes, hypercholesterolemia, current smoking, and serum creatinine level

*BMI* body mass index, *CAC* coronary artery calcification, *CACS* coronary artery calcium score, *CI* confidence interval, *OR* odds ratio, *RR* relative risk, *TyG* triglyceride glucose

## Discussion

The present study aimed to evaluate longitudinal change of CAC related to TyG index after considering baseline CAC status in asymptomatic adults. A number of studies reported that the TyG index was related to the risk of developing traditional CV disease and adverse clinical events. Recent cross-sectional data from Brazilian Cardioprotective Nutritional Program Trial showed the positive association of TyG index with metabolic and behavioral risk factors [27]. In the present study, we identified that the incidence of CAC progression was elevated with an increasing TyG index irrespective of baseline CACS in this study. A remarkable result was that high TyG index was significantly associated with an increased risk of CAC progression in subjects who had baseline CACS ≤ 100 after adjusting for traditional risk factors.

The homeostatic model assessment of IR (HOMA-IR) has been traditionally used to measure IR [28, 29]. However, it is required to measure insulin levels for calculating the HOMA-IR. Considering that insulin levels are not usually achieved during general health check-up in South Korea, calculating the HOMA-IR to estimate IR is somewhat inconvenient for this population. A number of previous studies identified that the TyG index is closely correlated with the HOMA-IR [9, 30]. Moreover, recent data reported that the value of the TyG index predicting IR was better than that of the HOMA-IR [8, 31]. Based on these evidences, TyG index has been considered as a simple and useful surrogate marker of IR in clinical practice.

Although several cross-sectional studies previously reported a significant relationship between the TyG index and CAC prevalence, longitudinal data have been limited in clinical practice. Recently, Park et al. observed that an elevated TyG index was related to an increased risk of CAC progression in 1175 subjects [32]; however, they evaluated the impact of the TyG index on CAC progression without consideration of the subjects’ baseline CACS. Atherosclerosis-related major CV events commonly occur even among subjects with low CV risk burden [33, 34]. The Multi-Ethnic Study of Atherosclerosis study suggested that the CACS was a strong predictor of adverse clinical outcomes and provided clinical information beyond traditional CV risk factors [4]. In particular, Blaha et al. reported excellent prognosis in the absence of CAC in 44,052 consecutive asymptomatic subjects during a mean follow-up of 5.6 ± 2.6 years [35]. Considering the incremental impact of CAC progression on an adverse clinical outcome [5], it is a substantial issue to identify independent predictors for CAC progression in clinical practice.

In the current study, CAC progression was observed in 13.0% of participants without CAC at baseline. Importantly, we found that a high TyG index has a strong association with an increased risk of CAC progression in this population after adjusting for confounding clinical factors. This relation of the TyG index with CAC progression was consistent in the baseline conditions of CACS of 1–10 and 11–100. However, there was no significant impact of the TyG index on CAC progression in participants with baseline CACS > 100. This result might be affected by the relatively small sample size of participants with CACS > 100, which was about 10.6% in our study. Thus, the significance of the TyG index for predicting CAC progression is still uncertain in individuals with heavy calcification at baseline. Further prospective studies with a large sample size are necessary to confirm this issue.

The mechanism underlying the relationship of the TyG index with atherosclerosis is unclear. However, previous studies revealed the pivotal role of IR in atherosclerosis progression via promoting apoptosis of macrophages, endothelial cells, and vascular smooth muscle cells [36–38]. Recently, data from Coronary Artery Risk Development in Young Adults (CARDIA) study revealed that transitions in metabolic risk occurred early in life and metabolic dysfunction is related to subclinical cardiovascular phenotypes including CAC and myocardial hypertrophy and dysfunction [39]. Park et al. suggested that TyG index was an effective marker for early detecting subclinical coronary atherosclerosis even in the absence of traditional CV risk factors [40]. However, considering the inconsistent results for the association of metabolic syndrome with ASCVD in diabetic patients [41, 42], further large prospective investigation for the clinical significance of TyG index in established diabetes should be necessary in diverse ethnic population.

There are some limitations to the present study. First, the KOICA registry was based on a relatively healthy population who underwent health check-ups in healthcare centres. The present study only included subjects who experienced at least two CAC scan examinations with available data on the TyG index and diabetic status from the KOICA registry. Thus, this study could not represent the characteristics of overall participants of KOICA registry and a potential selection bias might be present. Second, this was a retrospective study, which may be affected by unidentified confounders. Third, we only evaluated the association between the baseline TyG index and CAC progression; however, the mean change of the TyG index was very small: − 0.02 ± 0.48. Longitudinal consecutive changes of the TyG index during follow-up could not be confirmed. Fourth, the homeostatic model assessment of IR was not analysed because insulin levels were not measured in the KOICA registry. Fifth, some important data including physical activity, alcohol consumption, family history of disease were unavailable. Sixth, we could not control the effect of medications for hypertension, diabetes, and hyperlipidaemia on CAC progression because of the observational study design. Finally, the present study included only the Korean population. However, this study is unique in that we identified the predictive value of the TyG index for CAC progression according to the baseline CAC status in a large sample of asymptomatic adults.

## Conclusion

The TyG index is strongly associated with CAC progression after adjusting for confounding clinical variables, especially in adults without heavy CAC at baseline.

## Supplementary information

**Additional file 1: Table S1.** Association between clinical variables and CAC progression.

**Additional file 2: Table S2.** TyG index (per 1-unit increase) and the risk of CAC progression according to baseline CACS 100.

## Data Availability

The datasets used and analyzed during the current study are available from the corresponding author on reasonable request.

## Abbreviations

ASCVD: Atherosclerosis-related cardiovascular disease
BMI: Body mass index
CV: Cardiovascular
CAC: Coronary artery calcification
CACS: Coronary artery calcium score
CI: Confidence interval
HbA1C: Hemoglobin A1C
HDL-C: High-density lipoprotein cholesterol
KOICA: Korea Initiatives on Coronary Artery Calcification
LCL-C: Low-density lipoprotein cholesterol
OR: Odds ratio
RR: Relative risk
TyG: Triglyceride glucose
