# Supplementary material for: Triglyceride glucose index is an independent predictor for the progression of coronary artery calcification in the absence of heavy coronary artery calcification at baseline
Source: Cardiovasc Diabetol. 2020 Mar 16;19:34. doi: 10.1186/s12933-020-01008-5 (PMC7074986; doi:10.1186/s12933-020-01008-5)
Supplement: Supplementary file 1 — Additional file 1: Table S1. Association between clinical variables and CAC progression. [file 12933_2020_1008_MOESM1_ESM.docx]

**Table S1. Association between clinical variables and CAC progression**

| Variables | OR (95% CI) | P |
| --- | --- | --- |
| Age, pre-1 years increase | 1.04 (1.03−1.04) | <0.001 |
| Male | 1.82 (1.52−2.18) | <0.001 |
| BMI, per-1 kg/m^2^ increase | 1.03 (1.01−1.05) | 0.001 |
| Hypertension | 1.17 (1.06−1.30) | 0.002 |
| Diabetes | 1.20 (1.06−1.37) | 0.004 |
| Hyperlipidemia | 1.25 (1.13−1.38) | <0.001 |
| Current smoking | 1.18 (1.06−1.31) | 0.002 |
| Creatinine, per-1 mg/dL increase | 2.42 (1.76−3.32) | <0.001 |
| TyG index tertiles |  |  |
| I (lowest) | 1 | − |
| II | 1.26 (1.12−1.42) | <0.001 |
| III (highest) | 1.41 (1.25−1.60) | <0.001 |
| Baseline categorical CACS |  |  |
| CACS 0 | 1 | − |
| CACS 1–10 | 7.30 (6.42−8.31) | <0.001 |
| CACS 11–100 | 5.08 (4.50−5.74) | <0.001 |
| CACS >100 | 4.22 (3.62−4.93) | <0.001 |

*BMI* body mass index, *CAC* coronary artery calcification, *CACS* coronary artery calcium score, *CI* confidence interval, *LDL-C* low-density lipoprotein cholesterol, *OR* odds ratio.
