# Supplementary material for: Triglyceride glucose index is an independent predictor for the progression of coronary artery calcification in the absence of heavy coronary artery calcification at baseline
Source: Cardiovasc Diabetol. 2020 Mar 16;19:34. doi: 10.1186/s12933-020-01008-5 (PMC7074986; doi:10.1186/s12933-020-01008-5)
Supplement: Supplementary file 2 — Additional file 2: Table S2. TyG index (per 1-unit increase) and the risk of CAC progression according to baseline CACS 100. [file 12933_2020_1008_MOESM2_ESM.docx]

**Table S2. TyG index (per 1-unit increase) and the risk of CAC progression according to baseline CACS 100**

|  | OR (95% CI) | P |
| --- | --- | --- |
| CACS ≤100 |  |  |
| Model 1 | 1.76 (1.64−1.90) | <0.001 |
| Model 2 | 1.39 (1.09−1.76) | 0.007 |
| CACS >100 |  |  |
| Model 1 | 1.33 (1.09−1.62) | 0.004 |
| Model 2 | 1.34 (0.72−2.49) | 0.351 |

Models: 1 = unadjusted; 2 = adjusted for age, male sex, BMI, systolic and diastolic BP, the level of total cholesterol, triglyceride, HDL-C, LDL-C, glucose, and creatinine, and current smoking.

*BMI* body mass index, *BP* blood pressure, *CAC* coronary artery calcification, *CACS* coronary artery calcium score, *CI* confidence interval, *HDL-C* high-density lipoprotein cholesterol, *LDL-C* low-density lipoprotein cholesterol, *OR* odds ratio, *TyG* triglyceride glucose.
